# Encapsulation of *Eucalyptus largiflorens* Essential Oil by Mesoporous Silicates for Effective Control of the Cowpea Weevil, *Callosobruchus maculatus* (Fabricius) (Coleoptera: Chrysomelidae)

**DOI:** 10.3390/molecules27113531

**Published:** 2022-05-31

**Authors:** Asgar Ebadollahi, Jalal Jalali Sendi, William N. Setzer, Tanasak Changbunjong

**Affiliations:** 1 Department of Plant Sciences, Moghan College of Agriculture and Natural Resources, University of Mohaghegh Ardabili, Ardabil 5697194781, Iran; 2 Department of Plant Protection, Faculty of Agricultural Sciences, University of Guilan, Rasht 416351314, Iran; jjalali@guilan.ac.ir; 3 Department of Chemistry, University of Alabama in Huntsville, Huntsville, AL 35899, USA; wsetzer@chemistry.uah.edu; 4 Aromatic Plant Research Center, 230 N 1200 E, Suite 100, Lehi, UT 84043, USA; 5 Department of Pre-Clinic and Applied Animal Science, Faculty of Veterinary Science, Mahidol University, Nakhon Pathom 73170, Thailand

**Keywords:** fumigant persistence, mesoporous materials, encapsulation, toxicity

## Abstract

Although the use of synthetic chemicals is the principal method for insect pest management, their widespread application has led to numerous side effects, including environmental pollution and threats to human and animal health. Plant essential oils have been introduced as promising natural substitutes for synthetic insecticides. However, high volatility and/or low durability are the main limiting factors for essential oil application for control of insect pests. Accordingly, along with an evaluation of the fumigant toxicity of *Eucalyptus largiflorens* essential oil against the cowpea weevil, *Callosobruchus maculatus*, essential oil was nanoencapsulated by two mesoporous silicates, MCM-41 and zeolite 3A, to enhance fumigant persistence and toxicity. The chemical profile of essential oil was also analyzed through gas chromatographic-mass spectrometry. *E. largiflorens* essential oil showed significant concentration-dependent toxicity against insect pests; a concentration of 5.16 μL/L resulted in 100% mortality after 48 h. The toxicity of essential oil could be attributed to the presence of various insecticidal terpenes, such as spathulenol (15.6%), cryptone (7.0%), and 1,8-cineole (5.8%). Fumigant persistence was increased from 6 days to 19 and 17 days for pure and capsulated essential oil with MCM-41 and Zeolite 3A, respectively. The insect mortality also increased from 99 insects in pure essential oil to 178 and 180 insects in MCM-41 and Zeolite 3A encapsulated formulations, respectively. Therefore, the encapsulation of *E. largiflorens* essential oil by MCM- 41 and Zeolite 3A is a beneficial method for enhancing its persistence and toxicity against *C. maculatus*.

## 1. Introduction

The cowpea weevil, *Callosobruchus maculatus* (Fabricius) (Coleoptera: Chrysomelidae), is the damaging field-carry-to storage insect pest of various legumes, such as beans, chickpea, cowpea, lentil, and soybean [1,2]. A female *C. maculatus* lays more than 100 eggs within its 2- to 3-week life span, and larvae penetrate legume grains, causing weight loss, as well as reduction in seed viability and nutritional quality, making grains unsuitable for marketing, human, and animal consumption [3,4]. The economic injury of *C. maculatus* can reach 100% in untreated legume grains [5].

Although the use of synthetic insecticides is the main strategy for the management of *C. maculatus*, they can be toxic to non-target organisms and contaminate the air, crops, soil, and underground water [6]. Furthermore, the resistance of *C. maculatus* to several conventional carbamate, pyrethroid, organophosphate, and organochlorine pesticides was reported [7,8,9]. Accordingly, the introduction of low-risk efficient agents for the management of such pests is necessary.

The use of essential oils extracted from aromatic plants as low risk materials to manage of insect pests from different genera, families, and orders has recently been recommended [10,11,12]. The essential oils from different parts of aromatic plants, such as leaves, flowers, and stems, are mixtures of aromatic and aliphatic compounds, including terpenoids and phenylpropanoids [13]. Along with significant roles in pollinator attraction and plant–plant interactions, these secondary metabolites are coevolved in plants against herbivores [14]. There are also multiple modes of action against insect pests: inhibition of acetylcholinesterase activity; blockage of octopamine, gamma-amino butyric acid, and nicotinic acetylcholine receptors; disruption of the function of detoxifying enzymes, esterases, and glutathione *S*-transferases; and adverse effects on digestive enzymes, such as lipases, proteases, amylases, and glucosidases, as well as energy reservoir protein, glucose, and triglyceride contents [15]. Therefore, insect pests are less resistant to toxic exposure to plant essential oils [16]. The essential oils isolated from different species of *Eucalyptus* genus (Myrtales: Myrtaceae) are among the promising bioagents for the management of insect pests [17,18,19,20]. For example, the significant fumigant toxicity of two *Eucalyptus* essential oils, namely *E. lehmanni* (Schauer) Benth and *E. astringens* Maiden, against adults of *C. maculatus*, the lesser grain borer (*Rhyzopertha dominica* (F.)), and the rust-red flour beetle (*Tribolium castaneum* (Herbst)) was reported by Hamdi et al. [21]. Complete control was achieved with a concentration of 50 μL/L air of *E. lehmanni* essential oil after 48 h, which was attributed to its main component, 1,8-cineole (34.6%).

Despite the promising insecticidal efficiency of essential oils, their application is restricted because of high volatility and degradation with light, oxygen, and moderate temperature [22]. Controlled-release techniques have been recognized as means suitable for smaller quantities of insecticides, along with protection from environmental factors, which can be applied more efficiently [23]. Nanoencapsulation is a practical approach to enhance both durability and insecticidal activity of essential oils or any other active ingredients [24].

Mesoporous silicas with nanoscale dimensions have high specific surface areas (>1000 m^2^/g) based on regular arrangements of micropores [25]. For instance, the aluminosilicate crystal zeolite 3A, with narrow structural pores measuring 3 Å (0.3 nm), enable adsorption of molecules with a diameter larger than the pore openings [26]. MCM-41 (Mobil Composition of Matter No. 41) was the first mesoporous solid to be synthesized from aluminosilicate gels [27]. MCM-41 and zeolites from several mesoporous nanoparticles have been studied for bioapplications as a matrix for drug-controlled release [28,29]. Recently, there has been growing interest in the application of mesoporous nanoparticles, such as zeolites and MCM-41, ranging from adsorption to active ingredient delivery [30]. Nanoencapsulation of highly volatile fragrances, such as essential oils and their components, in zeolites and MCM-41 for controlled release and enhancement of their durability is one of the recent promising applications of mesoporous materials [31,32]. For example, the antibacterial activity of film nanocomposite made from zein film and cinnamon essential oil was significantly prolonged by loading the essential oil in MCM-41 for application in long-term packaging [33]. In another study, the fumigation persistence of essential oils isolated from *Thymus eriocalyx* (Ronniger) Jalas and *Thymus kotschyanus* Boiss and Hohen was increased by up to three times by loading in MCM-41. In addition, the mortality of the pest (two-spotted spider mite: *Tetranychus urticae* Koch) exposed to these essential oils was significantly increased [34].

The present study was conducted to evaluate the toxicity of *E. largiflorens* essential oil against *C. maculatus*. The main objective was to achieve encapsulation by two mesoporous materials, MCM-41 and Zeolite 3A, to intensify fumigant persistence and insecticidal efficiency of essential oil. The essential oil was also analyzed by gas chromatography—mass spectrometry to clarify its chemical profile.

## 2. Results

### 2.1. Chemical Profile of E. largiflorens Essential Oil

**Figure 1:**
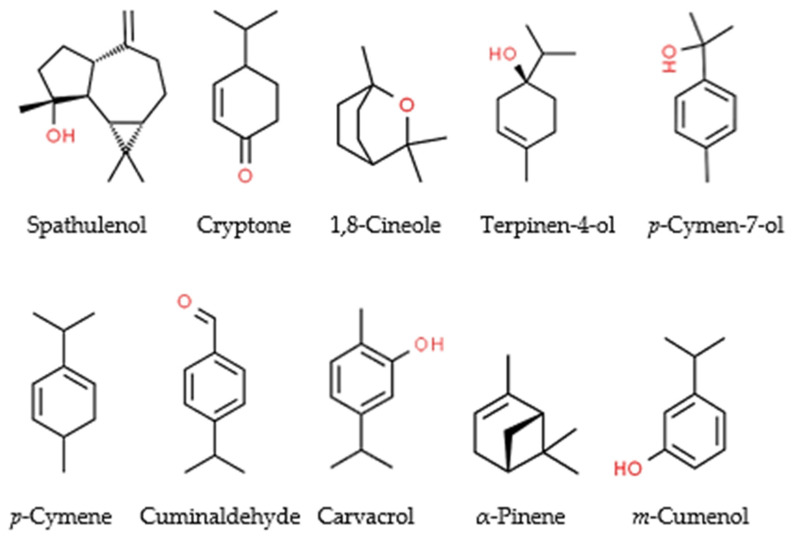
Chemical structure of main terpenes and terpenoids (highlighted with red O or OH) identified in the essential oil of *Eucalyptus largiflorens*.

Among 55 identified components, high levels of spathulenol (15.6%), cryptone (7.0%), 1,8-cineole (5.8%), terpinen-4-ol (5.7%), *p*-cymen-7-ol (5.1%), *p*-cymene (4.8%), cuminaldehyde (4.4%), carvacrol (3.9%), α-pinene (3.2%), and *m*-cumenol (3.1%) were detected (Figure 1 and Table 1). *E. largiflorens* essential oil was found to be rich in terpenic components. Different groups of terpenes, including oxygenated monoterpenoids (53.4%), oxygenated sesquiterpenoids (21.9%), monoterpene hydrocarbons (11.6%), and sesquiterpene hydrocarbons (2.5%), were identified in the essential oil. Only 2.5% of the recognized components were non-terpenes (Table 1).

### 2.2. Fumigant Toxicity

**Figure 2:**
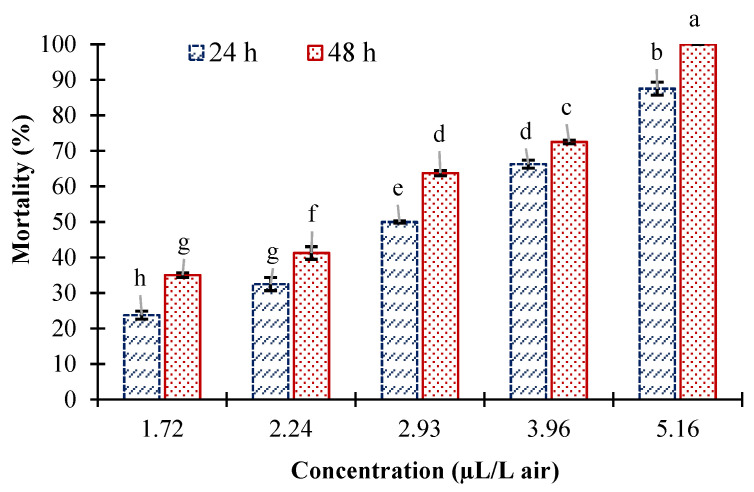
Mean mortality percentage (±SE) of *C. maculatus* adults exposed to fumigation with *Eucalyptus largiflorens* essential oil. Different letters indicate statistically significant differences according to Tukey’s test at *p* ≤ 0.05.

Data obtained from the fumigant toxicity of *E. largiflorens* essential oil against adults of *C. maculatus* had a normal distributions based on the result of a Kolmogorov–Smirnov test (Z = 0.67, 2-tailed significance = 0.75). According to the ANOVA, essential oil concentrations (F = 106.86, df = 4, and *p* < 0.001) and exposure times (F = 22.05, df = 1, and *p* < 0.001) had statistically significant effects on the mortality of *C. maculatus*. However, the interaction between these factors was not significant (F = 0.36, df = 4, and *p* < 0.833). The essential oil had prominent fumigant toxicity against *C. maculatus*; a fumigation concentration of 5.16 μL/L resulted in 100% mortality among the affected adults after 48 h. According to a comparison of means by Tukey’s test, increasing the concentration of essential oil and exposure time significantly augmented the mortality of insect pests (Figure 2).

The results of probit analyses of the data obtained from a fumigant toxicity assay of *E. largiflorens* essential oil against the adults of *C. maculatus*, including LC (lethal concentration) values, χ², and regression lines, are presented in Table 2. The LC_50_ (lethal concentration to kill 50% of tested insects) value of *E. largiflorens* essential oil on *C. maculatus* adults was 2.85 μL/L air after 24 h, which was decreased to 2.35 μL/L air after 48 h. However, this decrease was not statistically significant based on the overlapping of confidence limits. High values of *r*^2^ (correlation coefficients) also indicated a positive and direct correlation between essential oil concentrations and the mortality of *C. maculatus* for both exposure times (Table 2).

### 2.3. Encapsulation and Fumigant Persistence

**Figure 3:**
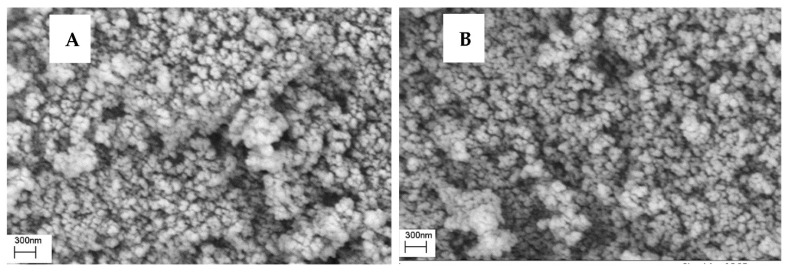

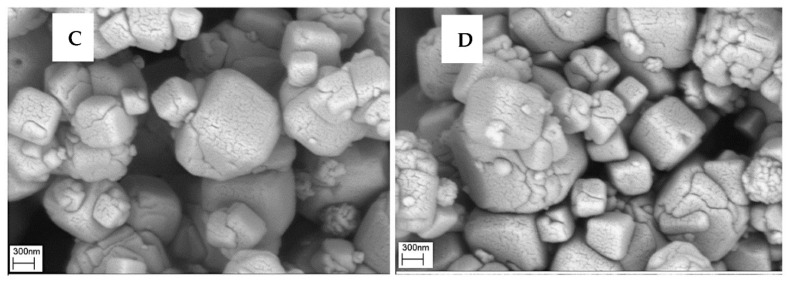
Scanning electron microscopy (SEM) micrographs ((**A**): MCM-41; (**B**): MCM-41-essential oil; (**C**): zeolite 3A; (**D**): zeolite 3A-essential oil).

**Figure 4:**
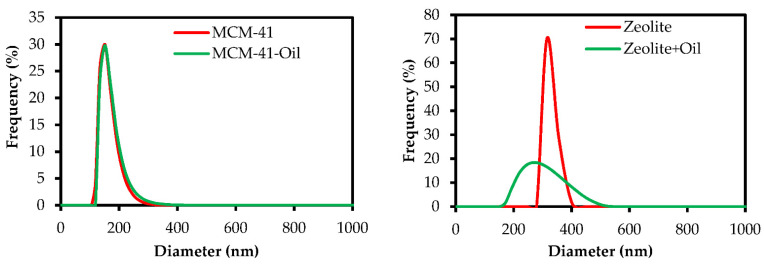
Dynamic light scattering (DLS) of MCM-41, MCM-41-essential oil, zeolite 3A, and zeolite 3A essential oil.

Nanoencapsulation efficiency percentage and loading percentage of *E. largiflorens* essential oil by MCM-41 were 81.25 and 79.12%, respectively. These values for zeolite 3A were 85.37 and 83.48%, respectively. The size of synthesized mesoporous particles evaluated by scanning electron microscopy (SEM) and dynamic light scattering (DLS) are presented in Figure 3A–D and 4; the zeolite 3A and MCM-41 with essential oil encapsulated treatment can be recognized as micro- and nanoparticles, respectively.

According to DLS analysis, the average particle sizes of MCM-41 and Zeolite 3A changed from 151 to 157 nm and 272 to 308 nm, respectively, as a result of loading of essential oil. The results of DLS analysis showed that the mean particle size of Zeolite 3A was larger than that of Zeolite 3A essential oil, and loading the essential oil slightly increased the particle size of MCM-41 (Figure 4).

**Figure 5:**
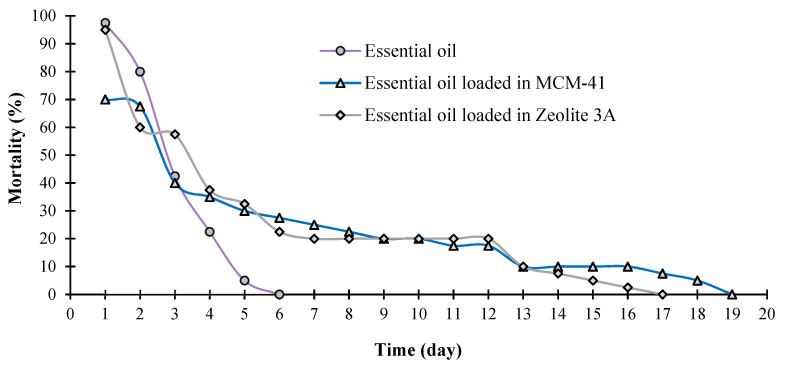
Fumigant toxicity and persistence of pure and nanoencapsulated *Eucalyptus largiflorens* essential oil against *Callosobruchus maculatus*. A concentration of 6.25 μL/L air (24-h LC_90_ value) was considered for both pure and nanoencapsulated oil. To eliminate the mortality in the control group (without any essential oil), the mortality percentage observed each day was corrected using Abbott’s formula.

Persistence of the essential oil of *E. largiflorens* was terminated after 6 days. Loading of *E. largiflorens* essential oil in MCM-41 and Zeolite 3A increased its persistence to 19 and 17 days, respectively. Although pure and capsulated essential oil formulations resulted in up to 50% mortality after 5 days, encapsulation of essential oil increased the efficiency of fumigant toxicity. Insect mortality increased from 99 insects in pure essential oil to 178 and 180 insects in MCM-41 and Zeolite 3A, respectively (Figure 5).

## 3. Discussion

Terpenic components, such as spathulenol, cryptone, 1,8-cineole, terpinen-4-ol, *p*-cymen-7-ol, *p*-cymene, cuminaldehyde, carvacrol, and α-pinene, were detected at high levels in the essential oil of *E. largiflorens*. The essential oil composition of *E. largiflorens* in this investigation is qualitatively similar to that reported in previously published studies [38,39,40]. However, some notable quantitative differences include the relatively high concentrations of 1,8-cineole (23.1–47.0%) coupled with relatively low concentrations of spathulenol (0.4–2.7%) in the previous reports. Such differences may be due to several exogenous and endogenous factors, including harvesting time, geographical position, extraction method, and genetic makeup [19,41,42]. Insecticidal properties of some components identified in the essential oil of *E. largiflorens* have previously been reported (Table 3). Along with acute toxicity, these components caused several sublethal effects, including the inhibition of acetylcholine esterase, adenosine triphosphatases, female oviposition and F1 adult emergence, and even repellent activity, on the treated insect pests, which is indicative of their multiple modes of action (Table 3). In other words, there is a mixture of insecticidal compounds with various biological effects in *E. largiflorens* essential oil, which are probably responsible for the observed toxicity. However, synergistic and antagonistic properties of other compounds in the essential oil should be considered [43].

Micro- and nanoencapsulated formulations based on controlled-release techniques have recently been introduced to enhance the persistence and possible application of plant essential oils for pest management [54,55,56,57]. For example, the toxicity of gelatin-based microencapsulated essential oils of *Rosmarinus officinalis* L. and *Thymus vulgaris* L. against the larvae of Indian meal moth, *Plodia interpunctella* (Hubner), were reported [58]. It was found that 5% of microcapsules in the diet of larvae caused 17.5 and 20.0% mortality by *R. officinalis* and *T. vulgaris* essential oils after 7 days, respectively. In another study, fumigant toxicity and persistence of *Cuminum cyminum* L. essential oil and oil loaded in myristic acid-chitosan nanogels were extended against the granary weevil (*Sitophilus granarius* L.) and confused flour beetle (*Tribolium confusum* Jacquelin du Val) [59]. In the other research, pure and maltodextrin/Angum gum nanoencapsulated essential oil of *Eucalyptus globulus* Labill with high concentrations of terpenic compounds 1,8-cineol and *p*-cymene were toxic to third-instar larvae of the Mediterranean flour moth (*Ephestia kuehniella* Zeller). Encapsulation enhanced both the insecticidal activity and the persistence of *E. globulus* essential oil [24]. In the present study, significant fumigant toxicity and persistence of micro- and nanoencapsulated *E. largiflorens* essential oil based on mesoporous materials were attained against *C. maculatus* as new formulations. These formulations based on controlled-release techniques allowed for a smaller reduction in essential oil quantity over a given time interval, as in the abovementioned studies.

A few studies documented the encapsulation of plant-derived materials by mesoporous silica for augmentation of their bioeffects against detrimental agents, including fungi and insects. For example, the antifeedant activity of α-pinene and linalool (as two major constituents in the essential oils of many aromatic plants) was improved against the tobacco cutworm (*Spodoptera litura* F.) and the castor semilooper (*Achaea janata* L.) by loading in silica nanoparticles [60]. Bernardos et al. [61] demonstrated that antifungal activity of some nanoencapsulated essential oil components comprising carvacrol, cinnamaldehyde, eugenol, and thymol by MCM-41 was intensified against *Aspergillus niger* ATCC 6275 so that encapsulated carvacrol and thymol was able to maintain antifungal activity, even after 30 days. In our recent study, the persistence of essential oils isolated from *Thymus eriocalyx* (Ronniger) Jalas and *Thymus kotschyanus* Boiss and Hohen was modified by loading in MCM-41 from 6 and 5 days in pure formulation to 20 and 18 days in nanoencapsulated formulations, respectively [34]. In the present work, an outlook was established of the utilization of *E. largiflorens* essential oil by loading in MCM-41 and zeolite 3A for enhancement of its fumigant toxicity and efficiency against a key pest, *C. maculatus*.

Chemical compositions of plant essential oils normally comprise terpenic and phenylpropanoic components. In the present study, spathulenol (15.6%) was found to be the most abundant component in the essential oil of *E. largiflorens*, followed by cryptone (7.0%), 1,8-cineole (5.8%), terpinene-4-ol (5.7%), *p*-cymen-7-ol (5.1%), *p*-cymene (4.8%), and cuminaldehyde (4.4%). Essential oils comprise a mixture of several compounds that may be cause a reduction in pest resistance. Furthermore, multiple biochemical effects of pure essential oils have been reported, including inhibition of acetylcholinesterase (AChE), blockage of the octopamine and gamma-aminobutyric acid receptors (GABArs), and reduction in detoxifying enzyme (esterase and glutathione *S*-transferases (GSTs)) activities and energy resources against insect pests [62,63,64]. Another characteristic of pure essential oils, besides high insecticidal efficiency against a wide range of insect pests, is low mammalian toxicity [62,63]. Furthermore, their components, in general, are biodegradable, in contrast to their ecotoxicology [10,65]. These positive attributes highlight the promising efficiency of essential oils against insect pests. However, low persistence is the main factor restricting the application of essential oils as part of pest management strategies. In this study, the fumigant persistence of *E. largiflorens* essential oil was improved by encapsulation in MCM-41 and Zeolite 3A. Although both physical and chemical mechanisms may be considered in loading and releasing essential oils from porous materials, research is often focused on physical means due to the presence of several nanopores in these materials [31,66]. For example, Milićević et al. [67] indicated that zeolite adsorbs the droplets of clove bud essential oil due to its porosity; the insecticidal, antifungal, and antibacterial effects of essential oils were augmented by loading in this porous material. In a study by Hettmann et al. [68], a functionalized calcium carbonate porous coating was used for controlled release of rosemary and thyme essential oil. The authors found that thyme essential oil required almost double the time required by rosemary essential oil to reach the saturation point, which can be explained by diverse chemical composition and concentration of monoterpenes between the two essential oils. Essential oil evaporates from the pores of porous materials and distributes to the air. The study by Hettmann et al. [68] mainly focused on physical influences, including degrees of loading and temperature, on release of essential oils from porous materials.

## 4. Materials and Methods

### 4.1. Materials

All required materials, including aluminum oxide, anhydrous sodium sulphate (Na_2_SO_4_), aqueous ammonia (25% *w/w*), cetyl trimethyl ammonium bromide (CTAB), silicon dioxide, sodium acetate trihydrate, sodium aluminate, sodium orthosilicate, sodium oxide, and tetraethyl orthosilicate (TEOS), were obtained from Merck (Darmstadt, Germany).

### 4.2. Plant Materials

Leaves of *Eucalyptus largiflorens* were collected from Kashan Botanical Garden, Kashan (33°59′20″ N, 51°28′38″ E), Iran. Voucher samples were deposited at the herbarium of Kashan Botanical Garden and characterized by scientific name.

### 4.3. Essential Oil Extraction

The samples were air-dried at room temperature and ground with an electric grinder. Essential oils were extracted using a Clevenger apparatus with 100 g of plant samples and 1200 mL distilled water for 3 h, and additional water from extracted essential oil was removed with Na_2_SO_4_. Extracted essential oil was stored in a refrigerator at 4 °C prior to experiments.

### 4.4. Essential Oil Analysis

Chemical analysis of the essential oils was carried out using an Agilent 7890B series GC instrument coupled with an Agilent 5977A Series mass spectrometer (Santa Clara, CA, USA). The GC column was an HP-5ms fused-silica capillary column (30 m length, 0.25 mm diameter) with a film thickness of 0.25 μm. The following oven temperature program was initiated at 50 °C, held for 1 min, then increased to 100 °C at a rate of 8 °C/min, increased 2 °C/min to 110 °C and held at 110 °C for 2 min, increased 5 °C/min to 185 °C, increased 15 °C/min to 280 °C and held at 280 °C for 2 min, and then increased 10 °C/min to 300 °C and held at 300 °C for 5 min. The spectrometers were operated in EI mode; the scan range was 50–500 amu, the ionization energy was 70 eV, and the scan rate was 0.20 s per scan. The injector, interface, and ion source were kept at 280 °C. Helium was used as the carrier gas, with a 1.0 mL/min flow rate. The sample was prepared by diluting essential oil in in methanol (1:10), and 1 μL of the solution was injected. The relative percentages of each component present in the analyzed EOs were calculated from TIC areas. The retention indices were determined in relation to a homologous series of *n*-alkanes (C_8_–C_20_) under the same operating conditions. Compounds were identified by comparing mass spectral fragmentation patterns and retention indices with those obtained from mass spectral libraries [35,36,37].

### 4.5. Insect Rearing

The initial colony of *C. maculatus* was obtained from the Department of Plant Protection, Faculty of Agriculture and Natural Resources, University of Mohaghegh Ardabili. The insects were reared on cowpea seeds (*Vigna ungiculata* (L.): Nekador cultivar) for several generations. Unsexed pairs of insects were transferred to uncontaminated cowpea seeds and removed after 48 h. The seeds contaminated with insect eggs were kept separately in an incubator 27 ± 2 °C, 75 ± 5% relative humidity with as 12: 12 dark: light photo period. Adults (1–7 days old) were designated for the bioassays [69].

### 4.6. Fumigant Toxicity of Essential Oil

Glass containers (580-mL) with tight caps were used as fumigant chambers. Based on the preliminary experiments, five essential oil concentrations (1.72, 2.24, 2.93, 3.96, and 5.16 μL/L air) were tested against the adults of *C. maculatus* (20 insects/container). All concentrations were sprayed evenly using a handheld sprayer on a 2 × 2 cm strip of Whatman no. 1 filter papers adhered to the inner surface of the lid of fumigation chambers. The exposure period was 24 and 48 h, and four replications were made for each concentration plus the control group. The controls were prepared with the same procedures, without any essential oil concentration. Insects were considered dead when they did not respond to touch with a paintbrush. The estimated mortality was checked for normality with the Kolmogorov–Smirnov test [70]. The mortality percentage was corrected using Abbott’s formula to account for the mortality in the control group [71]: P_t_ = [(P_o_ _ P_c_)/(100 _ P_c_)] × 100, where P_t_ is the corrected mortality percentage, P_o_ is the mortality percentage of treated insects, and P_c_ is the mortality percentage in the control group. The mortality data obtained from a fumigant toxicity assay of essential oil were subjected to analyses of variance (ANOVA), and comparison of means was performed at *p* ≤ 0.05 using Tukey’s test. Probit analyses were performed to estimate LC_50_ and LC_90_ values (lethal concentration to kill 50 and 90% of tested insects, respectively), regression line details, and *χ*^2^ values. SPSS version 24.0 (Chicago, IL, USA) was used as statistical software.

### 4.7. Synthesis of Mesoporous Materials

Pure-silica MCM-41 was synthesized according to the procedure described by Ebadollahi et al. [34]. CTAB as template was dissolved in a mixture of water: ethanol (155:1), and an ammonia solution and sodium acetate were then added. After 10 min stirring at 200 rpm, when a clear solution was obtained, TEOS was immediately added. The molar ratio of the resulting gel was 1 TEOS, 0.22 CTAB, 0.034 sodium acetate, 11 NH_3_, 1 ethanol, and 155 water. After 2.5 h stirring at room temperature, the obtained gel was transferred to a stainless-steel vessel and held at 70 °C for 5 h. The resulting powder was collected using filtration and then washed with water. The resulting surfactant containing MCM-41 was dried at 70 °C for 3 h, and the synthesized powder was culminated at 550 °C for 5 h to remove the template phase.

In the synthesis of Zeolite A3, aluminum oxide, silicate, sodium oxide, and water were used at a ratio of 1, 2, 0.55, and 0.45 (0.55 Na_2_O, Al_2_O_3_, 2 SiO_2_, 0.45 H_2_O). The resulting mixture was poured into water and, after adding potassium chloride (35% zeolite weight), was stirred for 2 h. The mixture was passed through a filter and dried after washing.

### 4.8. Encapsulation of Essential Oil and Persistence Assays

A total of 20 mg of each mesoporous material was added to a solution of 100 µL acetone and 6.25 µL of *E. largiflorens* essential oil (considered as a LC_90_ value). The weight of mesoporous material (20 mg) was measured in the control group after 20 min shaking at room temperature. At the same time, the final encapsulated essential oil was prepared with the same approach, and all trials were carried out without essential oil in the control group. The size distributions of particles were determined by scanning electron microscopy (SEM) (Hitachi su8040) and a dynamic light scattering (DLS) (HORIBA model, Japan). The encapsulation efficiency percentage and loading percentage were determined as follows [72]:

$$\mathrm{Nanoencapsulation}\;\mathrm{Efficiency}\;\mathrm{Percentage}=\frac{\;\mathrm{weight}\;\mathrm{of}\;\mathrm{encapsulated}\;\mathrm{essential}\;\mathrm{oil}}{\mathrm{weight}\;\mathrm{of}\;\mathrm{essential}\;\mathrm{oil}\;\mathrm{used}\;\mathrm{initially}}\times 100$$

$$\mathrm{Loading}\;\mathrm{Percentage}=\frac{\;\mathrm{weight}\;\mathrm{of}\;\mathrm{encapsulated}\;\mathrm{essential}\;\mathrm{oil}}{\mathrm{weight}\;\mathrm{of}\;\mathrm{essential}\;\mathrm{oil}\;-\;\mathrm{encapsulated}\;\mathrm{particles}}\times 100$$

Pure and nanoencapsulated essential oil persistence was measured based on fumigant bioassay; impregnated filter papers were used for pure essential oil under the lid of the fumigation chambers, and nanoencapsulated essential oil persistence was determined by mesoporous materials in 6-cm diameter Petri dishes in the bottom of the chamber. The LC_90_ value of essential oil determined by fumigant bioassay (6.25 μL/L air) was used for both pure and capsulated essential oils. In the case of nanoencapsulated essential oil, unloaded nanoparticles were considered as a control group. The mortality was counted for each 24 h of exposure time, and 10 adults (1–7 days old, randomly selected among both males and females) were introduced to the fumigant chamber for each time step. Four replications were considered for each treatment and the control groups. Mortality of insects treated with pure essential oil and encapsulated with MCM-41 and Zeolite 3A were recorded until the 6th, 17th, and 19th days, respectively. The mortality percentage was corrected using Abbott’s formula [40] to correct for the mortality in the control group, and a comparison of means was performed at *p* ≤ 0.05 using Tukey’s test.

## 5. Conclusions

Micro- and/or nanoencapsulation and other controlled-release techniques were used to enhance the pesticidal effectiveness and persistence of plant essential oils. In this study, the essential oil of *E. largiflorens* was encapsulated in mesoporous materials MCM-41 and Zeolite 3A. All pure and capsulated essential oil formulations had significant fumigant toxicity against insect pests, with up to 50% mortality achieved after 5 days. Encapsulation of *E. largiflorens* essential oil improved its persistence from 6 days for pure essential oil to 19 and 17 days for capsulated formulation by MCM-41 and Zeolite 3A, respectively. Along with persistency increases, encapsulation of essential oil improved the effectiveness of fumigant toxicity, and insect mortality increased from 99 insects in pure essential oil to 178 and 180 insects in MCM-41 and Zeolite 3A encapsulated formulations, respectively. Accordingly, nano- and microencapsulation of *E. largiflorens* essential oil based on MCM-41 and Zeolite 3A improved efficiency against *C. maculatus*, resulting in improved residual toxicity compared to pure oil. However, additional studies are required to clarify the toxicity against other pests, as well as the residual effects of these agents on commodities and non-target organisms.

## Figures and Tables

**Table 1:** Chemical profile of the essential oil extracted from *Eucalyptus largiflorens* leaves.

RI_db_	RI_calc_	Compound	%	RI_db_	RI_calc_	Compound	%
932	932	α-Pinene	3.2	1239	1234	Carvone	0.4
969	970	Sabinene	0.3	1249	1244	Piperitone	1.4
974	974	β-Pinene	0.2	1273	1267	Phellandranal	2.6
988	988	Myrcene	0.1	1282	1277	(*E*)-Anethole	2.4
988	990	2,3-Dehydro-1,8-cineole	0.1	1289	1294	*p*-Cymen-7-ol	5.1
1002	1003	α-Phellandrene	0.1	1298	1303	Carvacrol	3.9
1022	1011	*m*-Cymene	0.4	1314	1325	4-Hydroxycryptone	0.9
1024	1023	*p*-Cymene	4.8	1346	1347	α-Terpinyl acetate	2.4
1024	1028	Limonene	1.1	1389	1386	β-Elemene	0.2
1026	1032	1,8-Cineole	5.8	1392	1396	(*Z*)-Jasmone	0.1
1054	1056	γ-Terpinene	0.8	1439	1441	Aromadendrene	0.9
1065	1062	*cis*-Sabinene hydrate	0.1	1458	1462	*allo*-Aromadendrene	1.3
1067	1065	*cis*-Linalool oxide (furanoid)	0.3	1489	1486	β-Selinene	0.1
1083	1083	Diallyl disulfide	0.1	1491	1493	10,11-Epoxycalamenene	0.4
1089	1090	*p*-Cymenene	0.6	1564	1561	*epi*-Globulol	0.3
1098	1097	Linalool	0.6	1567	1569	Palustrol	0.6
1101	1102	*cis*-Thujone (α-Thujone)	0.4	1577	1581	Spathulenol	15.6
1112	1112	*trans*-Thujone (β-Thujone)	0.3	1590	1614	Globulol	1.7
1118	1119	*cis-p*-Menth-2-en-1-ol	0.9	1592	1616	Viridiflorol	0.5
1122	1120	α-Campholenal	0.4	1629	1628	*iso*-Spathulenol	0.9
1137	1137	*trans*-Sabinol	0.8	1640	1648	Caryophylla-4(12),8(13)-dien-5α-ol	0.3
1141	1141	Camphor	1.6	1652	1653	α-Cadinol	0.2
1148	1149	Menthone	0.9	1668	1670	14-Hydroxy-9-*epi*-(*E*)-caryophyllene	0.3
1160	1160	Pinocarvone	1.3	1741	1744	*iso*-Bicyclogermacrenal	1.0
1174	1175	Terpinen-4-ol	5.7			Monoterpene hydrocarbons	11.6
1183	1189	Cryptone	7.0			Oxygenated monoterpenoids	53.4
1186	1193	α-Terpineol	0.9			Sesquiterpene hydrocarbons	2.5
1204	1202	Verbenone	1.1			Oxygenated sesquiterpenoids	21.9
1215	1212	*trans*-Carveol	1.0			Others	2.7
1224	1224	*m*-Cumenol	3.1			Total identified	92.0
1238	1233	Cuminaldehyde	4.4				

RI_calc_ = retention index determined with respect to a homologous series of *n*-alkanes on a HP-5ms column; RI_db_ = retention index from the databases [35,36,37].

**Table 2:** Results of probit analyses of the data obtained from a fumigant toxicity assay of *Eucalyptus largiflorens* essential oil against the adults of *Callosobruchus maculatus*.

Time (h)	N	LC_50_ with 95% Confidence Limit (µL/L Air)	LC_90_ with 95% Confidence Limit (µL/L Air)	χ^2^ (df = 3)	Slope	Intercept	Significance	r^2^
24	480	2.85 (2.65–3.07)	6.25 (5.43–7.64)	3.07	3.76	−1.71	0.38 *	0.97
48	480	2.35 (1.15–3.19)	4.82 (3.46–36.18)	18.57	4.15	−1.55	0.0003 **	0.95

N is the number of tested insects at each time point. * Because the significance level is greater than 0.05, no heterogeneity factor was used in the calculation of fiducial limits. ** Because the significance level is less than 0.05, a heterogeneity factor was used in the calculation of fiducial limits.

**Table 3:** Recently reported insecticidal effects of some chemical components existing in *E. largiflorens* essential oil.

Compound	Insecticidal Activity
Anethole	Toxicity and acetylcholine esterase inhibitory against German cockroach (*Blattella germanica* (L.)) [44].
1,8-Cineole	Toxicity, along with oviposition and F1 adult emergence, inhibitory against *C. maculatus* [45].
Camphor	Toxicity against larvae of cotton leaf worm (*Spodoptera littoralis* Boisduval) [43].
Carvacrol	Toxicity against mushroom fly (*Lycoriella ingenua* (Dufour)) [46].
Carveol	Toxicity and acetylcholine esterase inhibitory against *B. germanica* [44].
Cuminaldehyde	Toxicity against the larvae of *L. ingenua* [46].
Limonene	Toxicity against adults of the housefly (*Musca domestica*) [47].
*p*-Cymen-7-ol	Toxicity against *T. castaneum* and *L. serricorne* [48].
*p*-Cymene	Toxicity, repellency, and inhibition of acetylcholinesterase and adenosine triphosphatases on *T. castaneum* [49].
Phellandranal	Toxicity, along with acetylcholine esterase, inhibitory against *B. germanica* [44].
Pinocarvone	Toxicity against Japanese termite (*Reticulitermes speratus* Kolbe) [50].
Spathulenol	Toxicity against the aphid *Metopolophium dirhodum* (walker) and relatively non-toxic to non-target ladybird and earthworm [51]
Terpinen-4-ol	Toxicity and repellency against booklouse (*Liposcelis bostrychophila* Badonnel), cigarette beetle (*Lasioderma serricorne* F.), and *T. castaneum* [52].
Verbenone	Toxicity against *L. bostrychophila*, *L. serricorne* and *T. castaneum* [53].
α-Pinene	Toxicity, repellency, and inhibition of acetylcholinesterase and adenosine triphosphatases on *T. castaneum* [49].
α-Terpinyl acetate	Toxicity against *M. domestica* adults [47].

## Data Availability

The data that support the findings of this study are available upon reasonable request.

## Sample Availability

Samples of the compounds are not available from the authors.

## References

[1] Kalpna H.Y.A., Kumar R. (2022). Management of stored grain pest with special reference to *Callosobruchus maculatus*, a major pest of cowpea: A review. Heliyon.

[2] Hamdi S.H., Abidi S., Sfayhi D., Dhraief M.Z., Amri M., Boushih E., Hedjal-Chebheb M., Larbi K.M., Ben Jemâa J.M. (2017). Nutritional alterations and damages to stored chickpea in relation with the pest status of *Callosobruchus maculatus* (Chrysomelidae). J. Asia Pac. Entomol..

[3] Nwosu L.C., Ikodie G. (2021). Comparative study on the bionomics of *Callosobruchus maculatus* Fabricius (Coleoptera: Chrysomelidae) in cowpea varieties (host) and common bean varieties (non-host): Findings revealed important food security information. Int. J. Trop. Insect Sci..

[4] Oke O.A., Akintunde E.M. (2013). Reduction of the nutritional values of cowpea infested with *Callosobruchus maculatus* (Coleoptera: Bruchidae). Int. J. Agri. Sci..

[5] Obembe O.M., Ojo D.O., David Ileke K. (2020). Efficacy of *Kigelia africana* Lam. (Benth.) leaf and stem bark ethanolic extracts on adult cowpea seed beetle, [*Callosobruchus maculatus* Fabricius (Coleoptera: Chrysomelidae)] affecting stored cowpea seeds (*Vigna unguiculata*). Heliyon.

[6] Tudi M., Daniel Ruan H., Wang L., Lyu J., Sadler R., Connell D., Chu C., Phung D.T. (2021). Agriculture development, pesticide application and its impact on the environment. Int. J. Environ. Res. Public Health.

[7] Gbaye O.A., Oyeniyi E.A., Ojo O.B. (2016). Resistance of *Callosobruchus maculatus* (Fabricius) (Coleoptera: Bruchidae) Populations in Nigeria to Dichlorvos. Jordan J. Biol. Sci..

[8] Gbaye O.A., Holloway G.J. (2011). Varietal effects of cowpea, *Vigna unguiculata*, on tolerance to Malathion in *Callosobruchus maculatus* (Coleoptera: Bruchidae). J. Stored Prod. Res..

[9] Zongo S., Coulibaly A.Y., Drabo S.F., Gnankiné O., Kiendrebeogo M., Doumma A., Sembène M., Sanon A. (2021). Metabolic resistance to pyrethroids (Py) and organophosphates (Op) in *Callosobruchus maculatus* (fab.) (Coleoptera: Chrysomelidae: Bruchinae) a major pest of stored cowpeas in West Africa. Int. J. Pest Manag..

[10] Regnault-Roger C., Vincent C., Arnasson J.T. (2012). Essential oils in insect control: Low-risk products in a high-stakes world. Annu. Rev. Entomol..

[11] Isman M.B., Grieneisen M.L. (2014). Botanical insecticide research: Many publications, limited useful data. Trends Plant Sci..

[12] Pavela R., Benelli G. (2016). Essential oils as ecofriendly biopesticides? Challenges and constraints. Trends Plant Sci..

[13] Sharmeen J.B., Mahomoodally F.M., Zengin G., Maggi F. (2021). Essential Oils as natural sources of fragrance compounds for cosmetics and cosmeceuticals. Molecules.

[14] Divekar P.A., Narayana S., Divekar B.A., Kumar R., Gadratagi B.G., Ray A., Singh A.K., Rani V., Singh V., Singh A.K. (2022). Plant secondary metabolites as defense tools against herbivores for sustainable crop protection. Int. J. Mol. Sci..

[15] Ebadollahi A., Jalali Sendi J., Ziaee M., Krutmuang P. (2021). Acaricidal, insecticidal, and nematicidal efficiency of essential oils isolated from the *Satureja* genus. Int. J. Environ. Res. Public Health.

[16] Isman M.B. (2006). Botanical insecticidal, deterrents, and repellents in modern agriculture and increasingly regulated world. Annu. Rev. Entomol..

[17] Patiño-Bayona W.R., Nagles Galeano L.J., Bustos Cortes J.J., Delgado Ávila W.A., Herrera Daza E., Suárez L.E.C., Prieto-Rodríguez J.A., Patiño-Ladino O.J. (2021). Effects of essential oils from 24 plant species on *Sitophilus zeamais* Motsch (Coleoptera, Curculionidae). Insects.

[18] Demeter S., Lebbe O., Hecq F., Nicolis S.C., Kenne Kemene T., Martin H., Fauconnier M.-L., Hance T. (2021). Insecticidal activity of 25 essential oils on the stored product pest, *Sitophilus granarius*. Foods.

[19] Ben Jemaa M.J., Haouel S., Bouaziz M., Khouja M.L. (2012). Seasonal variations in chemical composition and fumigant activity of five *Eucalyptus* essential oils against three moth pests of stored dates in Tunisia. J. Stored Prod. Res..

[20] Barbosa L.C.A., Filomeno C.A., Teixeira R.R. (2016). Chemical variability and biological activities of *Eucalyptus* spp. essential oils. Molecules.

[21] Hamdi S.H., Hedjal-Chebheb M., Kellouche A., Khouja M.L., Boudabous A., Ben Jemaa J.M. (2015). Management of three pests’ population strains from Tunisia and Algeria using *Eucalyptus* essential oils. Ind. Crops Prod..

[22] Campos E.V.R., Proenc P.L.F., Oliveira J.L., Bakshi M., Abhilash P.C., Fraceto L.F. (2019). Use of botanical insecticides for sustainable agriculture: Future perspectives. Ecol. Indic..

[23] Huang B., Chen F., Shen Y., Qian K., Wang Y., Sun C., Zhao X., Cui B., Gao F., Zeng Z. (2018). Advances in targeted pesticides with environmentally responsive controlled release by nanotechnology. Nanomaterials.

[24] Emamjomeh L., Imani S., Talebi Jahromi K., Moharramipour S. (2022). Nanoencapsulation enhances the contact toxicity of *Eucalyptus globulus* Labill and *Zataria multiflora* Boiss essential oils against the third instar larvae of *Ephestia kuehniella* (Lepidoptera: Pyralidae). Int. J. Pest Manag..

[25] Lehmana S.E., Larsen S.C. (2014). Zeolite and mesoporous silica nanomaterials: Greener syntheses, environmental applications and biological toxicity. Environ. Sci. Nano.

[26] Gabrus E., Nastaj J., Tabero P., Aleksandrzak T. (2015). Experimental studies on 3A and 4A zeolite molecular sieves regeneration in TSA process: Aliphatic alcohols dewatering–water desorption. Chem. Eng. J..

[27] Narayan R., Nayak U.Y., Raichur A.M., Garg S. (2018). Mesoporous silica nanoparticles: A comprehensive review on synthesis and recent advances. Pharmaceutics.

[28] Vallet-Regi M., Ramila A., del Real R.P., Perez-Pariente J. (2001). A new property of MCM-41: Drug delivery system. Chem. Mater..

[29] Santos H.A., Salonen J., Bimbo L.M., Lehto V.P., Peltonen L., Hirvonen J. (2015). Mesoporous materials as controlled drug delivery formulations. J. Drug. Deliv. Sci. Technol..

[30] Niculescu V.C. (2020). Mesoporous silica nanoparticles for bio-applications. Front. Mater..

[31] Li Z., Huang J., Ye L., Lv Y., Zhou Z., Shen Y., He Y., Jiang L. (2020). Encapsulation of highly volatile fragrances in Y zeolites for sustained release: Experimental and theoretical studies. ACS Omega.

[32] Poyatos-Racionero E., Guarí-Borràs G., Ruiz-Rico M., Morellá-Aucejo Á., Aznar E., Barat J.M., Martínez-Máñez R., Marcos M.D., Bernardos A. (2021). Towards the enhancement of essential oil components’ antimicrobial activity using new zein protein-gated mesoporous silica microdevices. Int. J. Mol. Sci..

[33] Liu X., Jia J., Duan S., Zhou X., Xiang A., Lian Z., Ge F. (2020). Zein/MCM-41 nanocomposite film incorporated with cinnamon essential oil loaded by modified supercritical CO_2_ impregnation for long-term antibacterial packaging. Pharmaceutics.

[34] Ebadollahi A., Jalali Sendi J., Aliakbar A. (2017). Efficacy of nanoencapsulated *Thymus eriocalyx* and *Thymus kotschyanus* essential oils by a mesoporous material MCM-41 against *Tetranychus urticae* (Acari: Tetranychidae). J. Econ. Entomol..

[35] Adams R.P. (2007). Identification of Essential Oil Components by Gas Chromatography/Mass Spectrometry.

[36] Mondello L. (2016). FFNSC 3.

[37] NIST (2017). NIST17.

[38] Assareh M.H., Jaimand K., Rezaee M.B. (2007). Chemical Composition of the essential oils of six *Eucalyptus* species (Myrtaceae) from South West of Iran. J. Essent. Oil Res..

[39] Sefidkon F., Assareh M.H., Abravesh Z., Barazandeh M.M. (2007). Chemical composition of the essential oils of four cultivated *Eucalyptus* species in Iran as medicinal plants (*E. microtheca*, *E. spathulata*, *E. largiflorens* and *E. torquata*). Iran J. Pharm. Res..

[40] Rahimi-Nasrabadi M., Nazarian S., Farahani H., Fallah Koohbijari G.R., Ahmadi F., Batooli H. (2013). Chemical composition, antioxidant, and antibacterial activities of the essential oil and methanol extracts of *Eucalyptus largiflorens* F. Muell. Int. J. Food Proper.

[41] Lima A., Arruda F., Medeiros J., Baptista J., Madruga J., Lima E. (2021). Variations in Essential oil chemical composition and biological activities of *Cryptomeria japonica* (Thunb. ex L.f.) D. Don from different *Geographical Origins*—A critical review. Appl. Sci..

[42] Ben Marzoug H.N., Romdhane M., Lebrihi A., Mathieu F., Couderc F., Abderraba M., Khouja M.L., Bouajila J. (2011). *Eucalyptus oleosa* essential oils: Chemical composition and antimicrobial and antioxidant activities of the oils from different plant parts (stems, leaves, flowers and fruits). Molecules.

[43] Pavela R. (2014). Acute, synergistic and antagonistic effects of some aromatic compounds on the *Spodoptera littoralis* Boisd. (Lep., Noctuidae) larvae. Ind. Crops Prod..

[44] Yeom H.-J., Kang J.S., Kim G.-H., Park I.-K. (2012). Insecticidal and acetylcholine esterase inhibition activity of Apiaceae plant essential oils and their constituents against adults of German cockroach (*Blattella germanica*). J. Agric. Food Chem..

[45] Abeywickrama K., Adhikari A.A.C.K., Paranagama P., Gamage C.S.P. (2006). The efficacy of essential oil of *Alpinia calcarata* (Rosc.) and its major constituent, 1, 8-cineole, as protectants of cowpea against *Callosobruchus maculatus* (F.) (Coleoptera: Bruchidae). Canadian J. Plant Sci..

[46] Park I.-K., Kim J.N., Lee Y.-S., Lee S.-G., Ahn Y.-J., Shin S.-C. (2008). Toxicity of plant essential oils and their components against *Lycoriella ingenua* (Diptera: Sciaridae). J. Econ. Entomol..

[47] Zhang Z., Xie Y., Wang Y., Lin Z., Wang L., Li G. (2017). Toxicities of monoterpenes against housefly, *Musca domestica* L. (Diptera: Muscidae). Environ. Sci. Poll. Res..

[48] Liang J., Shao Y., Wu H., An Y., Wang J., Zhang J., Kong W. (2021). Chemical constituents of the essential oil extracted from *Elsholtzia densa* and their insecticidal activity against *Tribolium castaneum* and *Lasioderma serricorne*. Foods.

[49] Saad M.M.G., El-Deeb D.A., Abdelgaleil S.A.M. (2019). Insecticidal potential and repellent and biochemical effects of phenylpropenes and monoterpenes on the red flour beetle, *Tribolium castaneum* Herbst. Environ. Sci. Pollut. Res. Int..

[50] Seo S.M., Kim J., Kang J., Koh S.H., Ahn Y.J., Kang K.S., Park I.K. (2014). Fumigant toxicity and acetylcholinesterase inhibitory activity of 4 Asteraceae plant essential oils and their constituents against Japanese termite (*Reticulitermes speratus* Kolbe). Pestic. Biochem. Physiol..

[51] Benelli G., Pavela R., Drenaggi E., Desneux N., Maggi F. (2020). Phytol,(E)-nerolidol and spathulenol from *Stevia rebaudiana* leaf essential oil as effective and eco-friendly botanical insecticides against *Metopolophium dirhodum*. Ind. Crops Prod..

[52] Wang Y., Zhang L.T., Feng Y.X., Guo S.S., Pang X., Zhang D., Geng Z.F., Du S.S. (2019). Insecticidal and repellent efficacy against stored-product insects of oxygenated monoterpenes and 2-dodecanone of the essential oil from *Zanthoxylum planispinum* var. dintanensis. Environ. Sci. Poll. Res..

[53] Zhang Z., Pang X., Guo S., Cao J., Wang Y., Chen Z., Feng Y., Lei N., Du S. (2019). Insecticidal activity of *Artemisia frigida* Willd. essential oil and its constituents against three stored product insects. Rec. Nat. Prod..

[54] Maes C., Bouquillon S., Fauconnier M.-L. (2019). Encapsulation of essential oils for the development of biosourced pesticides with controlled release: A review. Molecules.

[55] Melanie M., Miranti M., Kasmara H., Malini D.M., Husodo T., Panatarani C., Joni I.M., Hermawan W. (2022). Nanotechnology-based bioactive antifeedant for plant protection. Nanomaterials.

[56] Abdollahdokht D., Gao Y., Faramarz S., Poustforoosh A., Abbasi M., Asadikaram G., Nematollahi M.H. (2022). Conventional agrochemicals towards nano-biopesticides: An overview on recent advances. Chem. Biol. Technol. Agric..

[57] López M.D., Cantó-Tejero M., Pascual-Villalobos M.J. (2021). New Insights into biopesticides: Solid and liquid formulations of essential oils and derivatives. Front. Agron..

[58] Sanna Passino G., Bazzoni E., Moretti M.D.L. (2004). Microencapsulated essential oils active against Indian meal moth. Bol. Sanid. Veg. Plagas.

[59] Ziaee M., Moharramipour S., Mohsenifar A. (2014). MA-chitosan nanogel loaded with *Cuminum cyminum* essential oil for efficient management of two stored product beetle pests. J. Pest Sci..

[60] Usha Rani P., Madhusudhanamurthy J., Sreedhar B. (2014). Dynamic adsorption of α-pinene and linalool on silica nanoparticles for enhanced antifeedant activity against agricultural pests. J. Pest Sci..

[61] Bernardos A.T., Marina P., Žáček É., Pérez-Esteve R., Martínez-Mañez M., Lhotka L., Kouřimská J., Pulkrábek Klouček P. (2015). Antifungal effect of essential oil components against *Aspergillus niger* when loaded in silica mesoporous supports. J. Sci. Food Agric..

[62] Oftadeh M., Sendi J.J., Ebadollahi A., Setzer W.N., Krutmuang P. (2021). Mulberry protection through flowering-stage essential oil of *Artemisia annua* against the lesser mulberry pyralid, *Glyphodes pyloalis* Walker. Foods.

[63] Liang J.Y., Yang Y.Y., An Y., Shao Y.Z., He C.Y., Zhang J., Jia L.Y. (2021). Insecticidal and acetylcholine esterase inhibition activity of *Rhododendron thymifolium* essential oil and its main constituent against two stored product insects. J. Environ. Sci. Health B.

[64] Blenau W., Rademacher E., Baumann A. (2012). Plant essential oils and formamidines as insecticides/acaricides: What are the molecular targets?. Apidologie.

[65] Isman M.B. (2020). Bioinsecticides based on plant essential oils: A short overview. Z. Für Nat. C.

[66] Jin L., Teng J., Hu L., Lan X., Xu Y., Sheng J., Song Y., Wang M. (2019). Pepper fragrant essential oil (PFEO) and functionalized MCM-41 nanoparticles: Formation, characterization, and bactericidal activity. J. Sci. Food Agric..

[67] Milićević Z., Krnjajić S., Stević M., Ćirković J., Jelušić A., Pucarević M., Popović T. (2022). Encapsulated clove bud essential oil: A new perspective as an eco-friendly biopesticide. Agriculture.

[68] Hettmann K., Monnard F.W., Melo Rodriguez G., Hilty F.M., Yildirim S., Schoelkopf J. (2022). Porous coatings to control release rates of essential oils to generate an atmosphere with botanical actives. Materials.

[69] Khani A., Asghari J. (2012). Insecticide activity of essential oils of *Mentha longifolia*, *Pulicaria gnaphalodes* and *Achillea wilhelmsii* against two stored product pests, the flour beetle, *Tribolium castaneum*, and the cowpea weevil, *Callosobruchus maculatus*. J. Insect Sci..

[70] Lilliefors H.W. (1967). On the Kolmogorov-Smirnov test for normality with mean and variance unknown. J. Am. Stat. Assoc..

[71] Abbott W.S. (1925). A method for computing the effectiveness of an insecticide. J. Econ. Entomol..

[72] Sansukcharearnpon A., Wanichwecharungruang S., Leepipatpaiboon N., Kerdcharoend T., Arayachukeata S. (2010). High loading fragrance encapsulation based on a polymer-blend: Preparation and release behavior. Int. J. Pharm..

